# Attenuation of *Pseudomonas aeruginosa* Quorum Sensing by Natural Products: Virtual Screening, Evaluation and Biomolecular Interactions

**DOI:** 10.3390/ijms21062190

**Published:** 2020-03-22

**Authors:** Lin Zhong, Vinothkannan Ravichandran, Na Zhang, Hailong Wang, Xiaoying Bian, Youming Zhang, Aiying Li

**Affiliations:** Helmholtz International Laboratory for Anti-Infectives, Shandong University-Helmholtz Institute of Biotechnology, State Key Laboratory of Microbial Technology, Shandong University, Qingdao 266237, China; 201511767@mail.sdu.edu.cn (L.Z.); vrvinothan@sdu.edu.cn (V.R.); 13342258573@163.com (N.Z.); wanghailong@sdu.edu.cn (H.W.)

**Keywords:** *Pseudomonas aeruginosa*, LuxR-type quorum sensing regulator, quorum sensing inhibitors, molecular docking, microscale thermophoresis, thermal shift assay

## Abstract

Natural products play vital roles against infectious diseases since ancient times and most drugs in use today are derived from natural sources. Worldwide, multi-drug resistance becomes a massive threat to the society with increasing mortality. Hence, it is very crucial to identify alternate strategies to control these ‘super bugs’. *Pseudomonas aeruginosa* is an opportunistic pathogen reported to be resistant to a large number of critically important antibiotics. Quorum sensing (QS) is a cell–cell communication mechanism, regulates the biofilm formation and virulence factors that endow pathogenesis in various bacteria including *P. aeruginosa.* In this study, we identified and evaluated quorum sensing inhibitors (QSIs) from plant-based natural products against *P. aeruginosa*. In silico studies revealed that catechin-7-xyloside (C7X), sappanol and butein were capable of interacting with LasR, a LuxR-type quorum sensing regulator of *P. aeruginosa*. In vitro assays suggested that these QSIs significantly reduced the biofilm formation, pyocyanin, elastase, and rhamnolipid without influencing the growth. Especially, butein reduced the biofilm formation up to 72.45% at 100 µM concentration while C7X and sappanol inhibited the biofilm up to 66% and 54.26% respectively. Microscale thermophoresis analysis revealed that C7X had potential interaction with LasR (*K*_D_ = 933±369 nM) and thermal shift assay further confirmed the biomolecular interactions. These results suggested that QSIs are able to substantially obstruct the *P. aeruginosa* QS. Since LuxR-type transcriptional regulator homologues are present in numerous bacterial species, these QSIs may be developed as broad spectrum anti-infectives in the future.

## 1. Introduction

Globally, multi-drug resistant infections kill almost 700,000 people per year and it is predicted that it would increase up to 10 million deaths per year by 2050 [1]. This is due to the misuse and overuse of existing antimicrobials in humans, animals and plants, resulting in the development of multi-drug resistant (MDR) pathogenic bacteria [2]. Conventional antibiotics target the fundamental processes of bacteria leading to growth arrest which in turn creates selective pressure and further multi-drug resistant variants [3]. World Bank estimated that the antimicrobial resistance (AMR) will not only be a health burden but also cause reduction in gross domestic production in 2050 that would be comparable to the 2008–2009 global financial crisis [4]. *Pseudomonas aeruginosa* is a Gram negative bacterium, common cause of pneumonia and infections especially in bloodstream, urinary tract and surgical-sites and burn wounds and plays vital roles in cystic fibrosis. *P. aeruginosa* was reported to be resistant to many antibiotics, including aminoglycosides, cephalosporins, fluoroquinolones, and carbapenems [5,6]. Hence, *P. aeruginosa* was placed on the top of priority pathogen list (critical) by World Health Organization for prioritizing the new antibiotic development against MDR pathogens [7].

Quorum sensing (QS) is a cell–cell communication mechanism controlled by releasing, sensing and responding to signal molecules called autoinducers (AIs) to regulate the biofilm formation, virulence, bioluminescence, and antibiotic production based on population density in both Gram-negative and Gram-positive bacteria [8]. Gram-negative bacteria use LuxI/R type QS systems that contain LuxI-type auto inducer synthases that lead to the production of acyl homoserine lactone (AHL) molecules as AIs and cognate LuxR-type receptor proteins. LuxR-type receptors are a class of transcriptional regulatory proteins that mediate QS pathways by interacting with AIs. LuxR-type protein such as LuxR, LasR, and TraR are highly unstable without AIs and the concentration of AIs will be proportionally low at low cell density [9]. Once the cell density increases, AI concentration also respectively increases. And these accumulated AHLs interact with LuxR receptor, leading to the LuxR-AHL complex stabilization. Subsequently, the LuxR-AHL complex binds to respective promoter region, and leads to the expression of downstream genes [10].

In *P. aeruginosa*, there are three well-established quorum sensing pathways reported so far, such as two LuxI/R-type systems (LasI/R and RhlI/R), a PQS (Pseudomonas quinolone signal) [11]. These systems are well-organized with LasI/R at the top of the hierarchy in the cascade. LasR consists of two independently folded domains: A larger ligand binding domain (LBD) at the amino-terminal and a smaller DNA-binding domain (DBD) at the carboxy-terminal [12]. When *N*-(3-oxododecanoyl)-l-homoserine lactone (3OC12-HSL), an AI of LasI/R system, interacts with LasR, it activates many downstream genes including the *lasI* synthase, and leads to the production of 3OC12-HSL. The LasR-3OC12-HSL complex also initiates the expression of QS systems by positively regulating *rhlR rhlI, pqsR,* and *pqsABCDE* genes. Once RhlR interacts with *N*-butanoyl-l-homoserine lactone (C4-HSL), it activates many genes, including those are essential for virulence factor production and biofilm formation [13]. 

The comprehensive understanding that *P. aeruginosa* and many other pathogens, control much of their virulence by QS, offered a new direction to a novel and robust “drug target” [14,15,16]. Many studies revealed that curbing QS would effectively reduce the biofilm formation and virulence [17,18,19]. Unlike conventional antibiotics, quorum sensing inhibitors (QSIs) will not kill the bacteria, rather they disarm the bacterial pathogenicity alone and hence they seem to have only slighter chances to get resistance [20]. Hence interfering with quorum sensing is considered to be a practicable and potential alternate approach especially in handling multi-drug resistant *P. aeruginosa* [21]. Consequently, drugs capable of interfering with QS are prone to escalate the susceptibility of the pathogenic bacteria towards host defense mechanisms and further clearance [22]. Natural products from plants have been playing vital roles against infectious diseases since ancient times and many drugs in use today are derived from plant natural sources. Here, in this study, we have screened LasR-specific QSIs from plant-derived natural products and evaluated their efficacy in suppressing QS-related phenotypes, including biofilm formation and virulence factors. Further we also studied bimolecular interactions of these QSIs with LasR protein using microscale thermophoresis and thermal shift assays.

## 2. Results

Plants and plant-based natural products have always been used as important sources for novel drug development against various diseases. Hence, we intended to identify and evaluate QSIs from plant-based natural products. 

### 2.1. Molecular Docking Studies

To screen QSIs against LasR, a transcriptional regulator of *P. aeruginosa* QS, virtual screening was performed using a natural product database of Chemfaces, China (http://www.chemfaces.cn) containing 4687 plant based natural products. 

**Figure 1:**
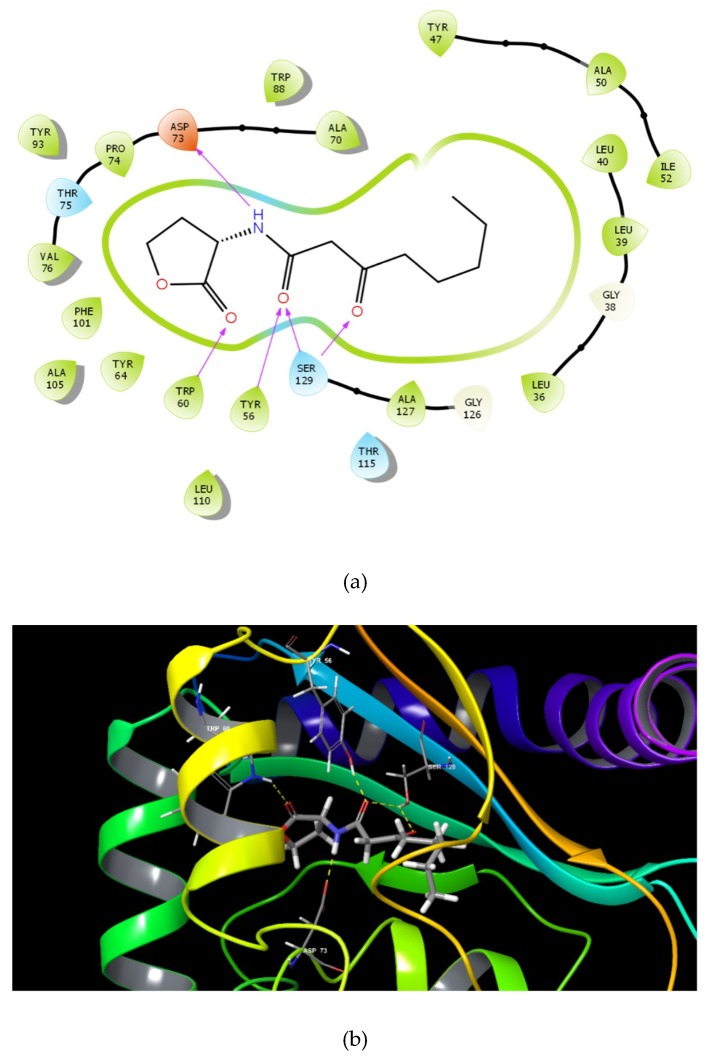
The 2D (**a**) and 3D (**b**) interaction map of 3OC12-HSL and LasR. Tyr 56, Trp 60, Asp 73 and Ser 129 (2).

Docking results showed that the GScore for the cognate ligand (3OC12-HSL) was -6.456 and it was able to form 5 H-bonds with Tyr 56, Trp 60, Asp 73, and Ser 129 (2) (Figure 1a,b). Pose analysis revealed that three compounds were able to interact with LasR. Considering the pattern of interaction of 3OC12-HSL, only compounds with similar pattern of interaction with significant GScore were chosen for further investigations. 

**Figure 2:**
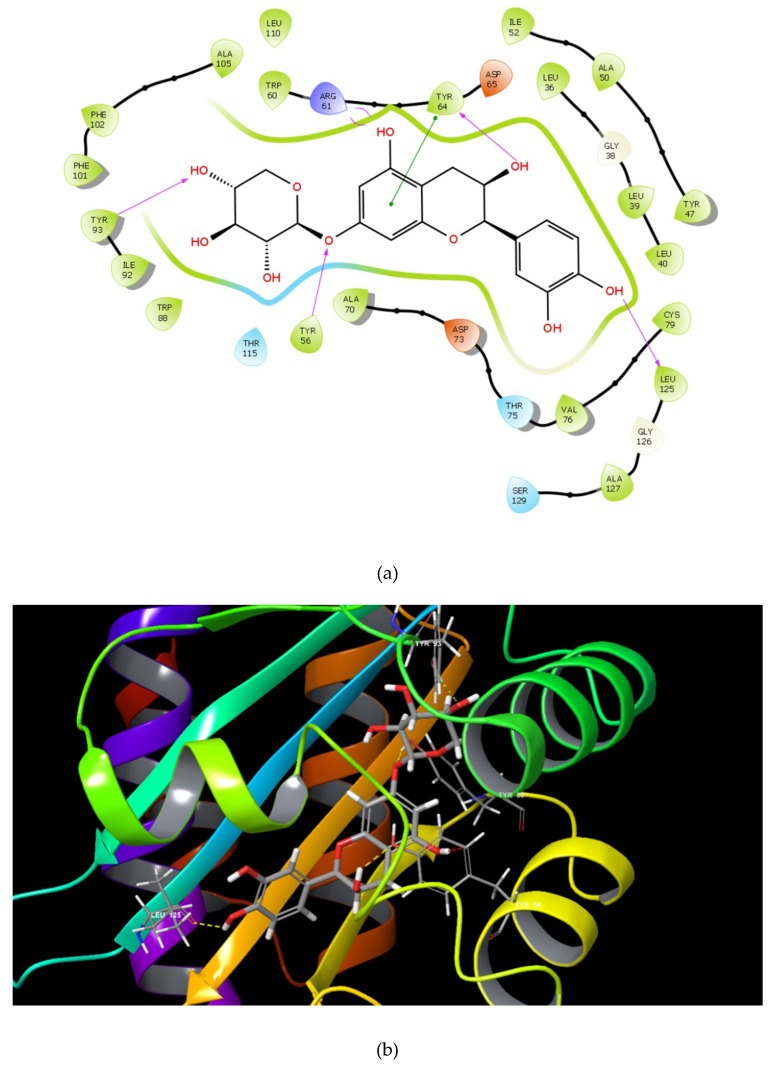
The 2D (**a**) and 3D (**b**) interaction map of C7X and LasR. Tyr 56, Tyr 64, Tyr 93, and Leu 125 are the interacting residues.

**Figure 3:**
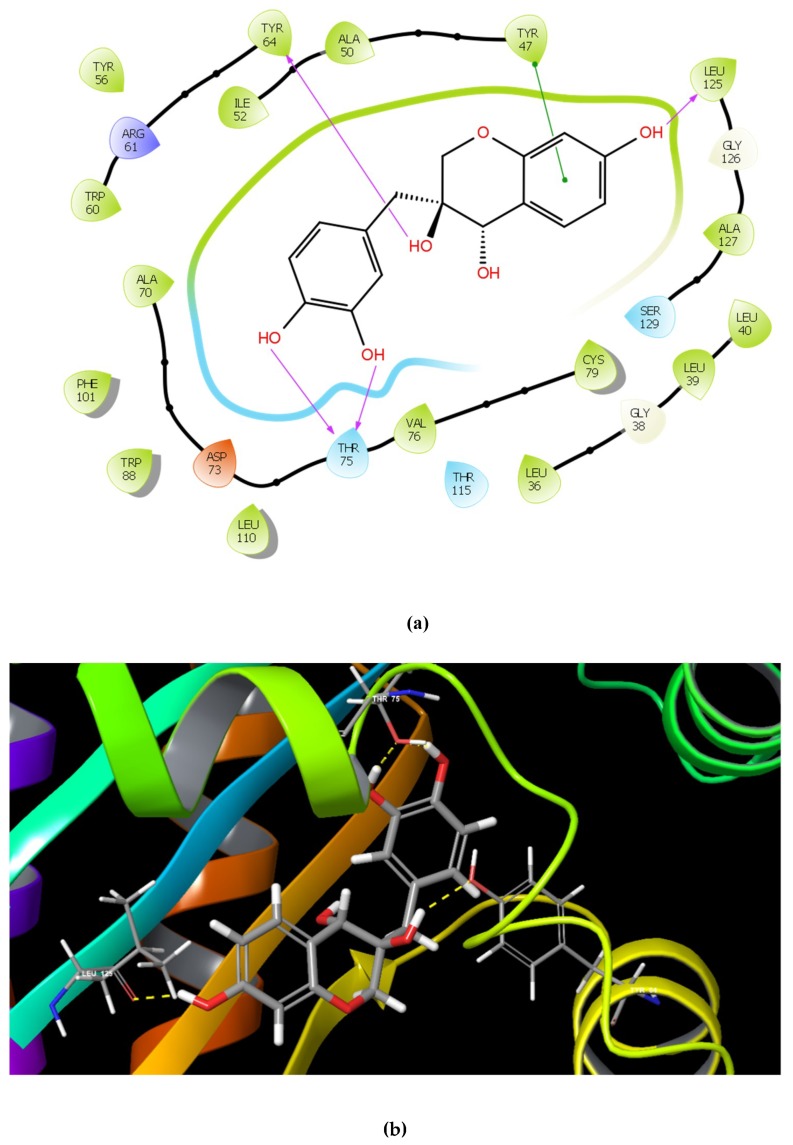
The 2D (**a**) and 3D (**b**) interaction map of sappanol with LasR. Tyr 64, Thr 75 (2), and Leu 125 are the interacting residues and Thr 75 is a key residue.

**Figure 4:**
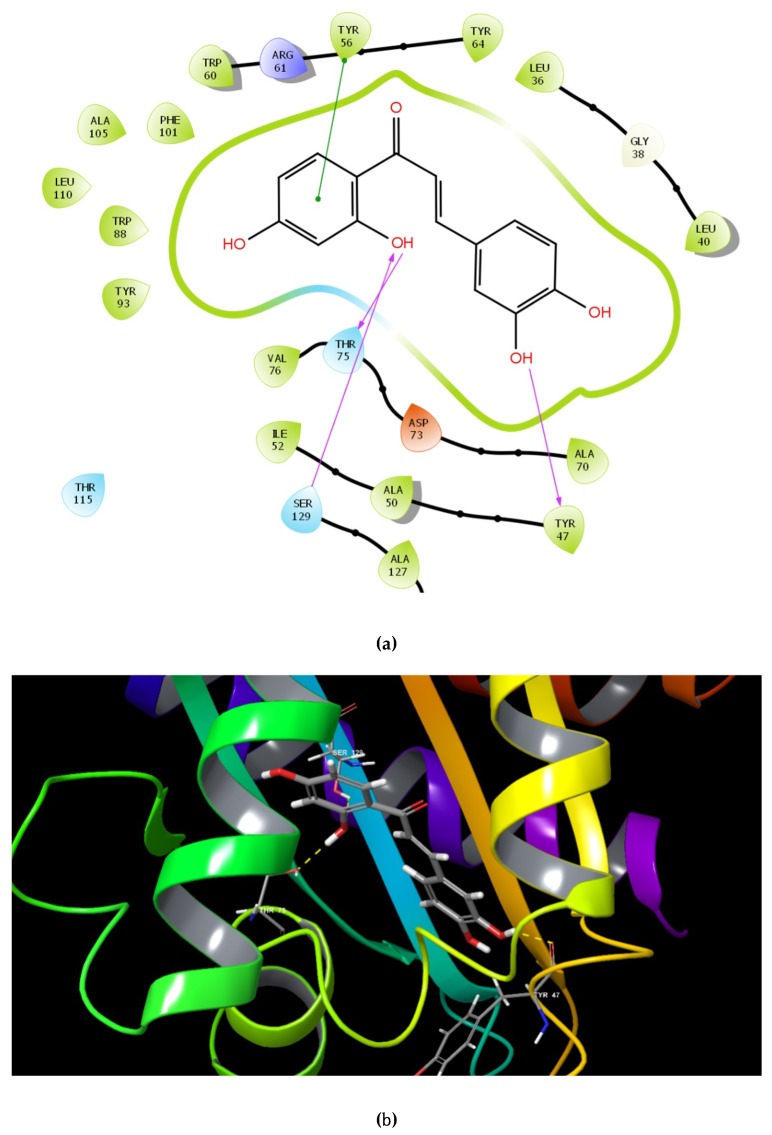
The 2D (**a**) and 3D (**b**) interaction map of butein and LasR. Tyr 47, Thr 75 and Ser 129 are the interacting residues. Tyr 47 was reported to be present in L3 loop crucial for the inhibitor binding.

**Figure 5:**
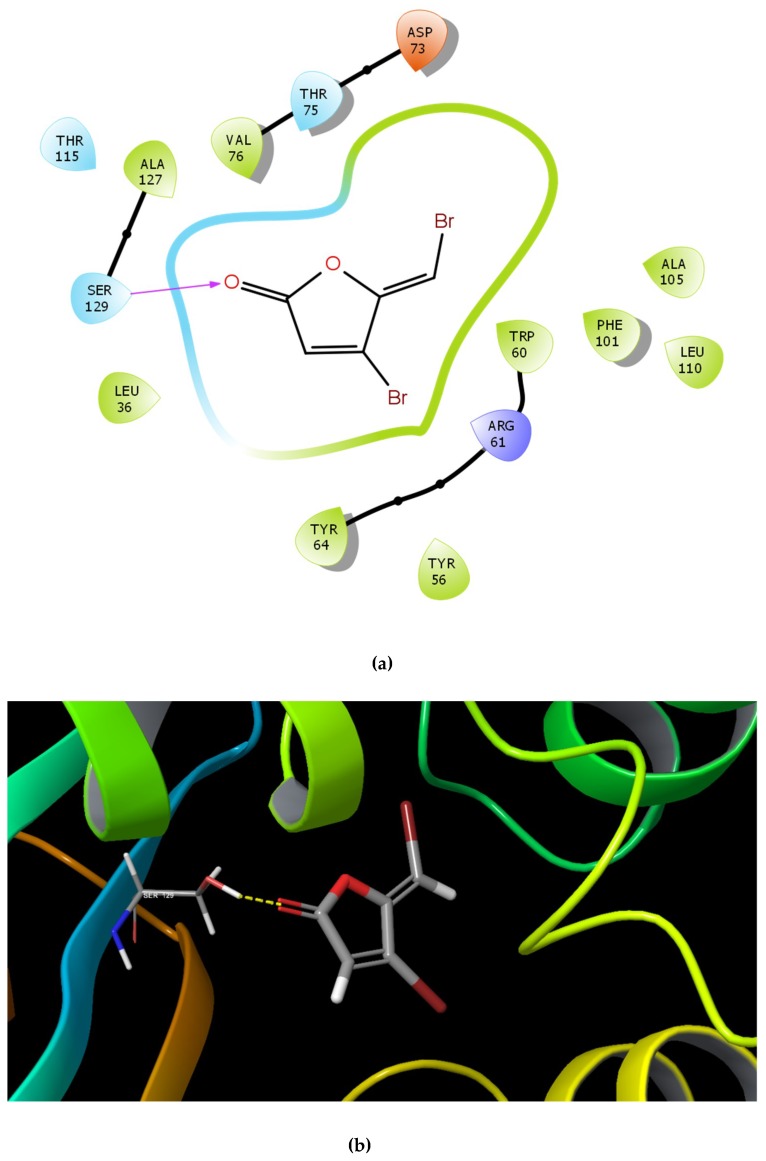
The 2D (**a**) and 3D (**b**) interaction map of C30 which interacting only with Ser 129.

The identified compounds C7X, sappanol, butein, and C30 ((Z-)-4-Bromo-5-(bromomethylene)-2(5H)-furanone) [16] were having the GScore −13.508, −10.964, −9.245 and −4.244 (Table 1) respectively. C7X was able to form 4 H-bonds at Tyr 56, Tyr 64, Tyr 93, Leu 125 and a pi–pi stalking with Tyr 64 (Figure 2a,b). Sappanol was able to form 4 H-bonds at amino acids Tyr 64, Thr 75 (2), Leu 125 and a pi–pi stalking with Tyr 47 (Figure 3a,b). Whereas, butein was able to form 3 H-bonds at Tyr 47, Thr 75, Ser 129 and a pi–pi interaction with Tyr 56 (Figure 4a,b). In contrast, C30 has only one H bond at Ser 129 (Figure 5a.b).

### 2.2. In vitro studies

#### 2.2.1. Influence on Biofilm Formation and Growth

**Figure 6:**
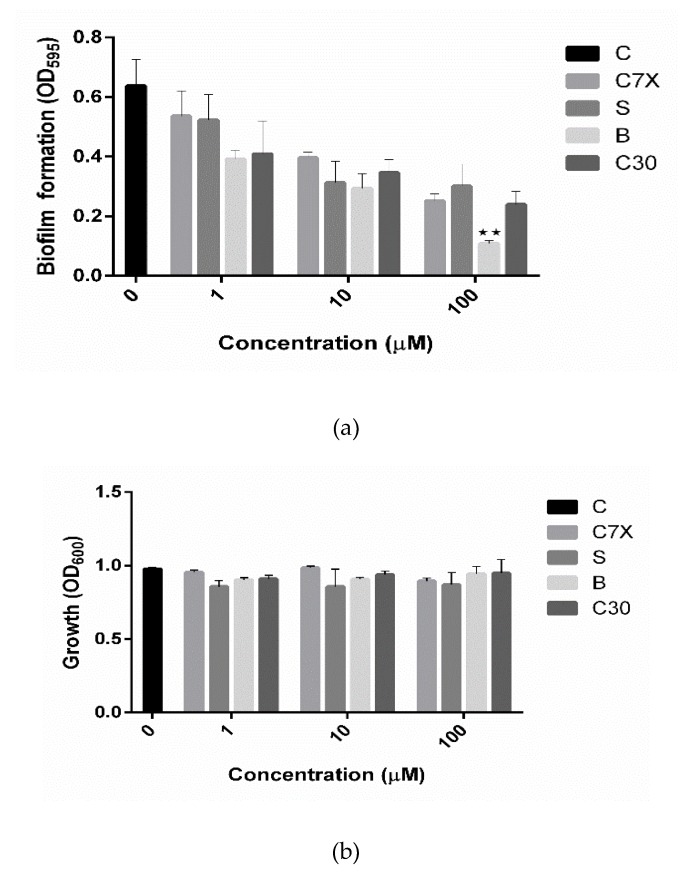
Effect of QSIs on biofilm and growth after 24 h (n = 3). (**a**) QSIs effect on biofilm. Butein (B) inhibited the biofilm formation significantly when compared to the untreated control (C). Data shown as mean ± SD of triplicate wells ** *p* < 0.01 vs. control; (**b**). None of the QSIs have affected the growth estimated by medial cloudiness. C is control, C7X, sappanol (S), butein (B) were QSIs and C30 is furanone, the known inhibitor. Data was statistically analyzed and the P-values were estimated by a student’s *t*-test.

Biofilm formation is one of the key factors which is controlled by QS and plays a fundamental role in pathogenesis [17,19]. All three compounds screened in this study were found to reduce the biofilm formation significantly at all the tested concentrations (1, 10 and 100 µM). All QSIs reduced the biofilm more than 50% at 100 µM concentration (Figure 6a). Especially, butein significantly reduced the biofilm formation about 72.45% with 100 µM concentration. While, C7X and sappanol reduced the biofilm about 66% and 54.26% respectively with 100 µM concentration. To differentiate the quorum sensing inhibition activity of these QSIs from antibiotic activity, the cell growth was also analyzed. It was found that none of QSIs had influence on the growth of *P. aeruginosa* (Figure 6b). 

#### 2.2.2. Confocal and Scanning Electron Microscope Studies

**Figure 7:**
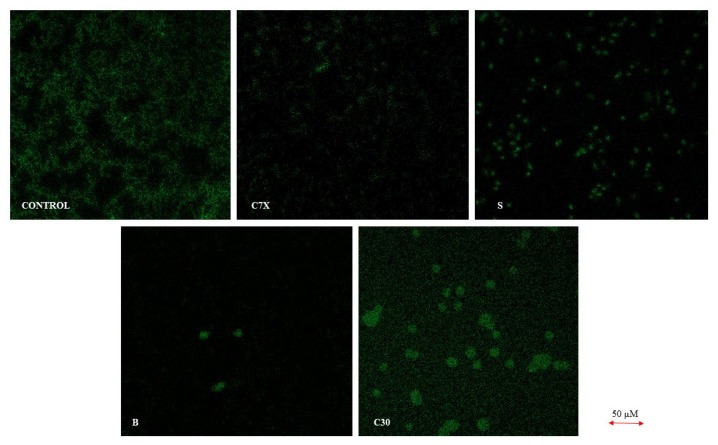
Biofilm inhibition by quorum sensing inhibitors (QSIs). Obvious biofilm inhibition was observed when treated with butein (B), C7X, C30 and sappanol (S) using FITC-CoA staining dye, compared to the control (C) where there is abundance of biofilm. Butein reduced the biofilm to the maximum when compared to the C30.

**Figure 8:**
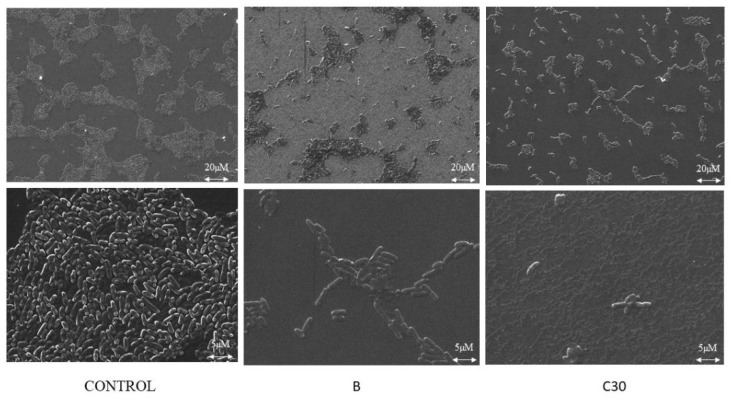
SEM analysis of biofilm inhibition. Butein (B) and C30 inhibited the biofilm formation. Obvious biofilm reduction was observed when treated with butein.

The biofilm inhibition was studied using the confocal laser scanning microscopy (CLSM). It was found that all the tested QSIs suppressed the biofilm formation when treated with 100 µM concentration (Figure 7). To be specific, butein significantly reduced the biofilm formation when administered with 100 µM. The scanning electron microscope (SEM) analysis showed a very similar pattern of inhibition which was confirmed by CLSM results. In control, we could see a lot of colonies in different size varying by compound to compound. All the QSIs were able to reduce the biofilm formation, especially butein was able to reduce the biofilm significantly equivalent to C30 (Figure 8). 

### 2.3. Influence on Virulence Factors

**Figure 9:**
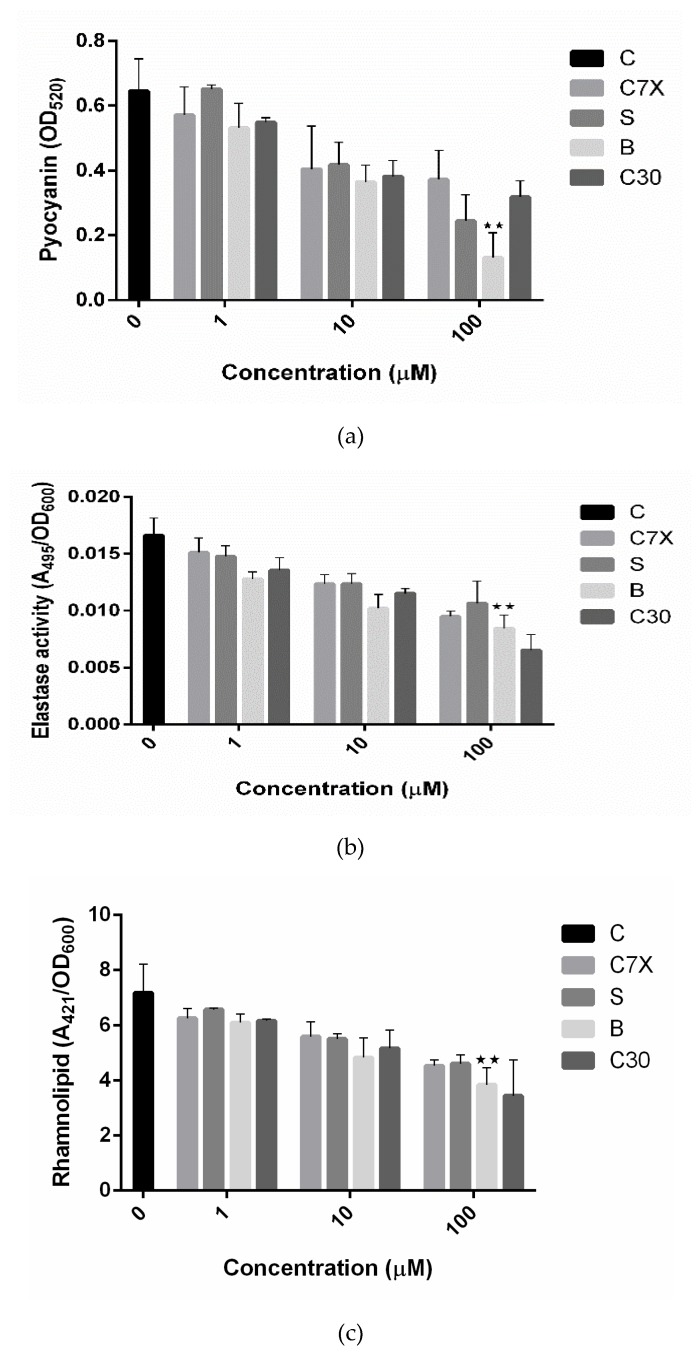
Effects of QSIs on virulence factors after 24 h (n = 3). (**a**) Pyocyanin production; (**b**) Elastase; (**c**) Rhamnolipid. Reduction in the QS-regulated virulence factors in the presence of butein (B) was observed. Butein reduced the pyocyanin production more significantly than C30, sappanol (S) and control (C). Data shown as mean ± SD of triplicate wells ** *p* < 0.01 vs. control.

Production of pyocyanin and rhamnolipid and elastase expression are also controlled by *P. aeruginosa*, and involved in its pathogenesis [17,19]. All the tested QSIs inhibited the pyocyanin production and especially butein showed 72.36% inhibition when treated with 100 µM concentration (Figure 9a). C30 also inhibited the pyocyanin production, comparable to that of C7X. Butein inhibited the elastase activity to about 33.98%, comparable to C30 (37.65%). C7X and sappanol also reduced the elastase activity significantly (Figure 9b). All the tested QSIs decreased the rhamnolipid production, especially butein which is comparable to that of C30 (Figure 7C). 

### 2.4. Inhibition on QS-Related Gene Expression

**Figure 10:**
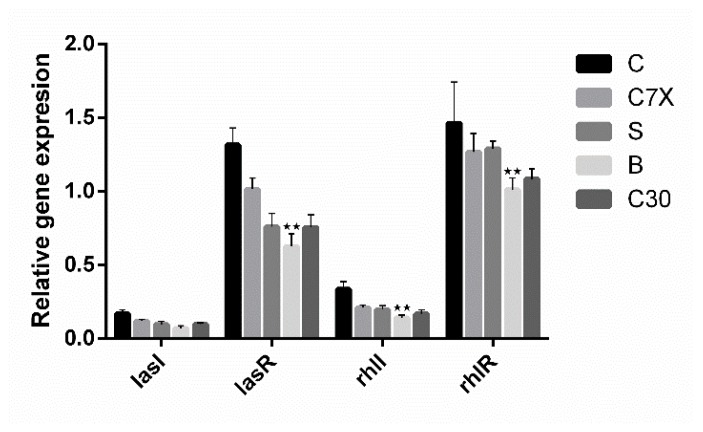
The influence of QSIs on the quorum sensing (QS) genes. qRT-PCR studies revealed that *lasR* and *rhlR* were inhibited significantly when compared to *lasI and rhlI* (*n* = 3). The level of QS-regulated gene expression was suppressed by QSIs especially by butein (B) (100 μM) when compared to the untreated control (C), sappanol (S) and C30. Data shown as mean ± SD of triplicates ** *p* < 0.01 vs. control.

To investigate the impact of QSIs (100 µM) on the genes related to the quorum sensing in *P. aeruginosa*, qRT-PCR studies were performed. The effect of QSIs on *lasI, lasR, rhlI, rhlR*, and *proC* genes was evaluated. Data suggested that the butein was able to significantly suppress the expression of *lasR* and *rhlR* (Figure 10), whereas *lasI* and *rhlI* were found to be comparatively less expressed.

### 2.5. Microscale Thermophoresis (MST) Analysis

For the LasR protein expression, we used RecET technology to clone the target directly. LasR-encoding gene *lasR* from genomic DNA of *P. aeruginosa* transferred into the expression vector and expressed successfully in *E. coli* (Figure S1). The purified LasR was used for MST measurement. MST experiment was carried out to investigate the bio-molecular interaction between LasR and QSIs. Variations in normalized fluorescence of the bound and unbound states will provide the fraction bound and thus the dissociation constant is calculated. All values are multiplied by a factor of 1000 to get the relative fluorescence change in per thousand. 

**Figure 11:**
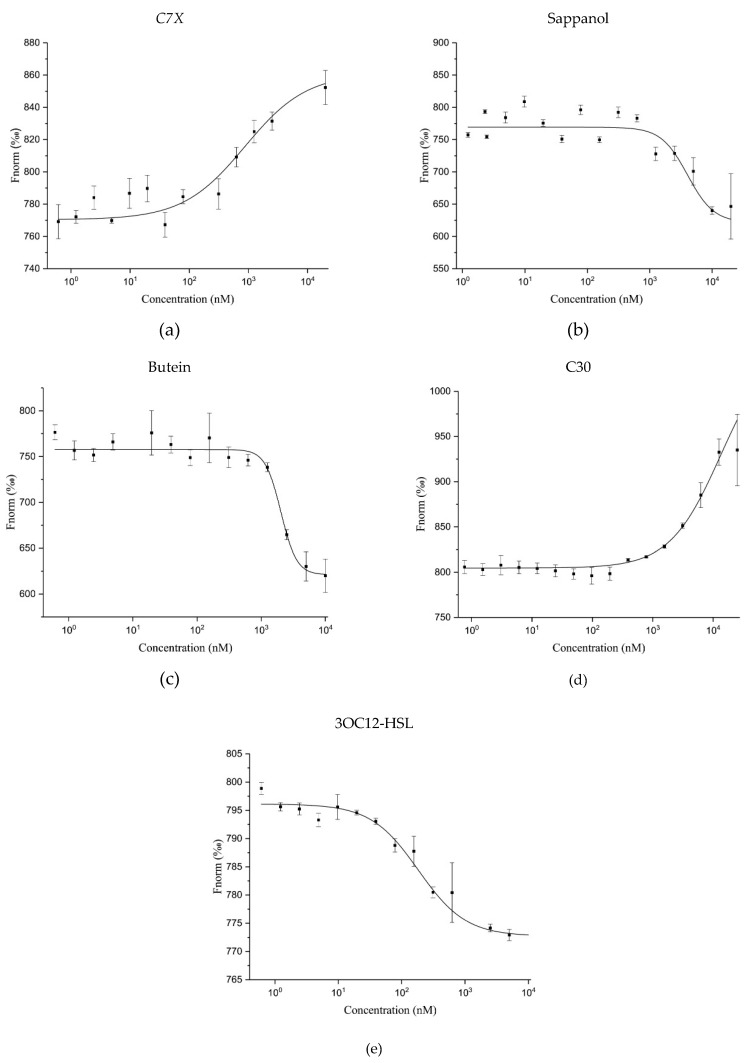
The molecular interaction of QSIs with LasR protein. (**a**) C7X binds to LasR with a *K*_D_ of 933±369; (**b**) Sappanol binds to LasR with a *K*_D_ of 5730±2870 nM; (**c**) Butein binds to LasR with a *K*_D_ of 3481±1631 nM; (**d**) C30 binds to LasR with a *K*_D_ of 20425±16003 nM; (**e**) 3OC12-HSL binds to LasR with a *K*_D_ of 271±58 nM.

MST results revealed that all the QSIs have significant binding ability with LasR. It is noteworthy to mention that the dissociation constant (*K*_D_) of C7X is 933 ± 369 nM which is comparatively lesser than other QSIs tested whereas the *K*_D_ of 3OC12-HSL is 271 ± 58 nM (Figure 11a,e). Surprisingly, the Fnorm value of sappanol and butein are in same downward trend with the fitted *K*_D_ of 5730 ± 2870 nM and 3481 ± 1631 nM respectively and the dose dependent downward trend is similar to that of 3OC12-HSL (Figure 11b,c) whereas C30 (*K*_D_ = 20425 ± 16003 nM) has a different upward trend in the normalized fluorescence. In upward trend the fluorescent molecules diffuse away from (positive thermophoresis) and in downward trend toward (negative thermophoresis) the heat focus. We speculate that upward trend of C7X and C30 is possibly caused by an irreversible binding mode. 

### 2.6. Thermal Shift Assay (TSA)

We conducted the thermal unfolding studies in the presence and absence of the inhibitors with a temperature gradient of 0.05 °C/s from 25 to 99 °C. No shift in ΔT occurred upon the addition of the DMSO as negative control solvent. Boltzmann Tm (Tm B) is the melting temperature (°C) calculated by fitting data in the region of analysis to the Boltzmann equation. 

**Figure 12:**
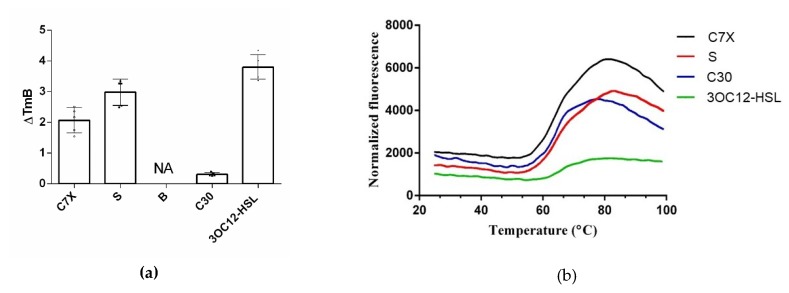
Thermal-shift analyses of purified LasR with QSIs. (**a**) Boltzmann Tm (Tm B) of the tested compounds. Data show that the interaction of sappnol (S) and C7X with LasR is comparatively strong as 3OC12-HSL-LasR interaction. No fluorescence was observed when treated with butein (B); (**b**) normalized fluorescence data represent a strong interaction with 3OC12-HSL-LasR, whereas the florescence revealed that the 3OC12-HSL-LasR interaction is strong enough to forbid the exposure of hydrophobic binding pocket where the dye used on this study supposed to bind.

A large shift in the Tm B (3.88 °C) (Figure 12a,b) occurred when 3OC12-HSL was added to LasR as positive control, indicating strong interaction between LasR and 3OC12-HSL. SYPRO orange seems to be normal, whereas Protein Thermal Shift™ Dye produces comparatively lower florescence suggesting strong interaction between LasR and 3OC12-HSL that in turn forbids the exposure of the hydrophobic region of LasR protein where the assay dye was supposed to bind (Figure 12a). C7X and sappanol has Tm B of 2.2 °C and 3.1 °C (Figure 12b) respectively suggesting a potential interaction with LasR.

## 3. Discussion

Plants have always been a great source for novel drug compounds, as plant derived medicines have made huge aids to human health. In this study we identified and evaluated three QSIs against *P. aeruginosa* from natural products database. Catechin-7-Xyloside (C7X) is flavan-3-ols, from *Spiraea hypericifolia*. Sappanol is a 3, 4-dihydroxyhomoisoflavan, from *Caesalpinia sappan*. Butein is a chalcone, from *Toxicodendron vernicifluum,* and C30 as a known inhibitor against *P. aeruginosa* [17], is (Z-)-4-Bromo-5-(bromomethylene)-2(5H)-furanone.

Targeting QS is the most extensively studied alternative strategy and demonstrated against various multi-drug resistant pathogens. Since QSIs curb several virulence factors and biofilm without affecting growth unlike antibiotics, it leads to the attenuation of pathogenesis while preventing selective pressure and development of antibiotic resistance [18]. Hence, QS interference seems to be a promising approach to address there MDR pathogens and can be developed as standalone anti-infective drugs or in combination with conventional antibiotics. 

Here, it is noteworthy to mention that docking results revealed butein was able to form H-bond with carbonyl oxygen of Tyr 47. McCready et al., 2018, reported that Tyr 47 present in loop L3 of LasR is able to adopt multiple conformations and hence it can accommodate the different ligands [23]. When LasR Tyr 47 was mutated with other amino acids such as Ser and Arg, the sensitivity to towards AHLs was found to be decreased. These results suggested that the interactions of the acyl side chain with the loop region promotes increased protein stability, in turn activates LasR. It is notable that the packing of Tyr 47 against the acyl chain protects the LBD from the bulk solvent and alterations at L3 loop region, leading to protein instability [12]. Also, LasR is agonized by ligands that were able to form H-bonds with Trp60 and Asp73 residues, and antagonized if they interacted with Tyr 47. 

Paczkowski et al., 2019 revealed that Thr 75, Tyr 93, and Ala 127 were able to convert low-potency QSIs into high-potency compounds and inactive ligands into low-potency QSIs by mutational analysis [24]. Here, we found that sappanol and butein have potential interaction with Thr 75. The acyl side chain has van der Waals interactions with the following hydrophobic pocket residues such as Leu 36, Leu 40, Tyr 47, Ile 52, Val 76, and Leu 125 at the end of the LasR LBD. These interactions seem to be crucial since they are involved in stabilization of the LasR–3OC12-HSL complex [25]. Our results also revealed that C7X and sappanol were able to interact with Leu 125. Overall docking results revealed that these compounds were able to interact with Tyr 47, Thr 75 and Leu 125, especially sappanol which interacts with both Thr 75 and Leu 125 whereas the butein is able to form H bonds with Tyr 47 and Thr 75. Thus our findings are in consistent with the earlier reports [25,26] suggesting that these residues which are mostly in hydrophobic region upon interactions with these QSIs may destabilize the LasR protein. 

Many natural products of plant origin were reported to be QSIs but their specificity towards LasR or any other quorum regulators has been poorly investigated. Ouyang et al., 2015 reported that the pyocyanin production was decreased by quercetin (16 μg/mL) by 58% [27]. Curcumin decreased the biofilm formation and decreased the elastase by 2 fold when administered with 1–3 μg/mL [28]. Vandeputte et al., 2010 reported that naringenin (4 mM) reduced the QS-regulated virulence factors especially pyocyanin and elastase [29]. Kim et al., 2015 found that the rhamnolipid production was suppressed by almost 36%–60% when they administered 6-Gingerol (0.1–100 μM) [30]. Paczkowski et al., 2017 identified that flavonoids were able to down regulate many virulence related traits, and especially flavonoids possessing dihydroxyl moieties such as baicalein and quercetin were able to inhibit QS by interacting with LasR [26]. It is noteworthy to mention that C7X and Sappanol are flavonoids, whereas butein is chalcone. Our study revealed that all the tested QSIs were able to reduce the biofilm, pyocyanin, elastase, and rhamnolipid production at the minimal concentration when compared to earlier studies. Numerous studies have been carried out in search of potential QSIs over phytochemicals sources, in which all the virulence related traits shown to be decreased [16,31,32]. Natural products including furonones, quercetin, naringenin, curcumin, catechin, were reported to be QSIs with varying activity range. Most of the studies were focused on measuring the biofilm and virulence phenotypes but no further efforts have been taken to evaluate mode of action and understand the biomolecular interactions of these QSIs the respective molecular targets. 

CLSM and SEM results revealed that these QSIs are able to reduce the biofilm which confirms our in vitro studies. Kim et al., 2015 also observed similar kind of inhibition using 6-gingerol [30]. Packiavathy et al., 2012 showed that eugenol inhibited the biofilm [33] and Sarabhai et al., 2013 showed that ellagic acid reduced the biofilm formation [32]. It is important to note that our study showed significant QSI effect at lower concentrations of phytochemicals when compared to aforesaid reports. Kim et al., 2015 showed that *lasR* and *rhlR* expression was drastically reduced by 6-gingerol (100 µM) [30]. It is found that quercetin (16 µg/mL) significantly reduced *lasI, lasR, rhlI,* and *rhlR* expression [23]. O’Loughlin et al., 2013 showed that mBTL decreased the QS-related genes [19]. In our study, it is observed that all the tested QSIs were able to influence the QS-regulated genes, especially butein significantly down-regulated *lasI*, *lasR, rhlI,* and *rhlR* genes. 

In order to affirm the specificity of these QSIs on LasR protein, we have performed MST and TSA. MST revealed that the tested QSIs were potential interaction with LasR protein. The strong interaction of C7X is coherent with our docking results since it has the better GScore with 4 H-bonds with Tyr 56, Tyr 64, Tyr 93, and Leu 125. Overall results of MST revealed the strong interaction of these QSIs with LasR. It is worthy to mention that sappanol and butein have a similar downward trend and they both were able to form H-bond Thr 75. Though C30 was able to form only one H-bond (Ser 123), it significantly curbed the QS pathway. We assume that C30 occupies the AHL binding pocket by interacting with Ser 129 which forbids 3OC12-HSL’s entry into the binding pocket. 

Paczkowski et al., 2017, reported the importance of Thr 75 in suppressing quorum sensing through interacting with LasR [26]. To our knowledge, this is the first report to use MST to study interaction between QSIs with LasR. The movement of molecules in a temperature gradient can be studied using MST, and the movement could be tracked by a fluorophore which can covalently interact with the protein. The interaction of proteins and ligands modifies this movement and thus MST can be used to detect binding ability of the molecules. The thermophoresis of a protein significantly differs from the thermophoresis of a protein-ligand complex as the binding induces fluctuations in size, charge and energy. Even if the interaction is not able to change the size or charge of a protein significantly, MST can still detect the binding due to binding-induced changes in the solvation entropy of the molecules. MST requires relatively low amounts of materials and that is advantageous over conventional instruments such as ITC and SPR [34]. 

TSA clearly indicates the biomolecular interaction of LasR and QSIs. Unfortunately, we found no fluorescence when treated with butein and it may be due to the interaction of butein with Tyr 47, a very crucial amino acid present in L3 loop. Since Tyr-47 is positioned in the L3 loop region which covers the LBD, the Tyr-47 interaction in the absence of the autoinducer would result in loop flexibility, and subsequent exposure of the hidden hydrophobic residues in LBD resulting in protein aggregation [35]. Compounds that induce inward moment of L3 loop with the bond length of 3.5–3.9Å found to be potential LasR inhibitors [12]. In MST we could not find any misfolding of LasR since the fluorescent dye, its interaction with protein and buffer system are entirely different from Thermal shift assay system. Hence docking results revealed the interaction between butein and Tyr 47 and the bond length of hydroxyl group of butein with carbonyl oxygen of Tyr 47 is 2.21Å (Figure S2), we assume that butein induce an inward moment of L3 loop of LasR. We also speculate that butein induces the movement of L3 loop leading to misfolding of LasR, in turn inducing protein aggregation. 

In our previous work [36], we have reported that C7X, sappanol and butein inhibited the QS mechanism in *Chromobacterium violaceum.* Though they might have different mode of interaction with CviR in *C. violaceum* from LasR in *P. aeruginosa,* their QS inhibition against both pathogenic bacteria implied that they might represent a broad spectrum anti-infective activity.

Structurally, Catechin-7-Xyloside (C7X) is flavan-3-ols, Sappanol is a 3, 4-dihydroxyhomoisoflavan, and butein is a chalcone. Chinese herbal products have been studied for many medical problems. The plants from which the QSIs have been identified are being used in traditional Chinese medicine (TCM) especially *Toxicodendron vernicifluum* used for the treatment of gastritis, stomach cancer, and atherosclerosis [37]. Paczkowski et al., 2017 revealed that flavonoids can potentially interact with LasR and suppress the quorum sensing in *P. aeruginosa* [26]. Among these QSIs, C7X, and sappanol are flavonoids whereas butein is a chalcone which showed comparatively significant quorum sensing inhibitory activity, implying structural diversity of potential QS-inhibitors.

## 4. Materials and Methods

### 4.1. Bacterial Strains and Growth Conditions

*Pseudomonas aeruginosa* (PAO1) was a wild type strain and used as test organism in this study and grown in Lysogeny Broth (LB) medium for 24 h at 37 ºC unless otherwise mentioned. Test compounds Catechin 7-xyloside (C7X), sappanol, and butein were purchased from Chemfaces, China (http://www.chemfaces.cn). *N*-(3-oxo-dodecanoyl)-l-homoserine lactone (3OC12-HSL), (Z-)-4-Bromo-5-(bromomethylene)-2(5H)-furanone (C30), and other chemicals were purchased from Sigma-Aldrich, (Shanghai, China) unless otherwise stated.

### 4.2. In Silico Studies

Molecular docking was performed using the Schrodinger suite software (Glide module V 8.2, New York, NY, USA) [38]. LasR protein (PDB 2UV0) was docked against natural products database. The ligands were prepared using LigPrep and the protein was processed further with Protein Preparation wizard. The receptor grid was generated based on 3OC12-HSL position in the crystal structure with the following residues present in the active site Tyr 56, Trp 60, Asp 73, and Ser 129. HTVS (High Throughput Virtual Screening) was performed to identify the QSIs and the XP (Extra Precision) was performed to further predict the interactions between these QSIs and LasR protein. 

### 4.3. In Vitro Assays

#### 4.3.1. Biofilm Inhibition Assay

Biofilm formation was measured using the microtitre plate assay to study the efficacy of QSIs [36,39]. Briefly, overnight cultures of *P. aeruginosa* PAO1 (OD600 nm = 0.4) were added into 1 mL of fresh LB medium with QSIs in different concentrations (1, 10, and 100 μM). Bacteria were grown up for 24 h at 37 ºC without any agitation. To remove the free-floating planktonic cells, the microtitre plate was washed with PBS (pH 7.4) after incubation. The biofilm was stained by adding 200 μL of crystal violet (0.1%) solution. Crystal violet solution was removed after 15 min and 200 μL of ethanol (95%) was added. The absorbance at OD470 nm was read using a microplate reader (Infinite M200, Tecan, Männedorf, Switzerland).

#### 4.3.2. Pyocyanin Production

From overnight cultures of *P. aeruginosa* PAO1 were grown with or without QSIs (1, 10, and 100 μM) in LB broth at 37 °C for 24 h. Then, the supernatants were collected, and the pyocyanin was extracted by using chloroform, followed by the addition of 0.2 M HCl. The crude extract obtained appears to be a pink solution, and the absorbance at OD520 nm was read [30].

#### 4.3.3. Elastase Activity

The elastolytic activity was measured using elastin congo red (ECR) as substrate. Briefly, the *P. aeruginosa* PAO1 were grown in LB broth at 37 °C for 24 h with or without QSIs (1, 10, and 100 μM). After the incubation period, centrifuged at 15,000 g at 4 °C for 10 min and then the resultant supernatant (0.5 mL) was added to 1 mL of assay buffer (30 mM Tris buffer, pH 7.2) which contains 10 mg of ECR. The reaction mixture was then incubated for 6 h at 37 °C with agitation. Centrifugation at 1200 g for 10 min was done to remove insoluble substrate and absorbance of the supernatant was measured at OD495 nm [40].

#### 4.3.4. Rhamnolipid Assay

Rhamnolipid was measured as previously reported with minor modifications [41]. The overnight culture of *P. aeruginosa* PAO1 was inoculated in LB medium with or without QSIs (1, 10 and 100 μM) and incubated at 37 °C for 24 h with agitation. After incubation, the cultures were centrifuged at 12,000 g at 4 °C for 5 min after 24 hrs. 500 μL of cell supernatants were added to 1 mL of diethyl ether (100%) and then the ether fraction was evaporated. The resultant fraction was eluted with 500 μL ddH2O, and then 100 μL of the subsequent eluting fraction was mixed with 900 μL orcinol solution (0.19% orcinal in 53% H_2_SO_4_). The reaction mixture was boiled for 30 min, and then cooled for 15 min at RT. Rhamnolipid production was estimated by measuring OD421nm in a spectrophotometer. 

### 4.4. Confocal Laser Scanning Microscopy Analysis

Confocal Laser Scanning Microscopy (CLSM) analysis of the *P. aeruginosa* PAO1 biofilms was performed as reported earlier [42]. Briefly, *P. aeruginosa* PAO1 was inoculated (1: 100) in LB broth on the cover slips to form static biofilms and incubated overnight at 37 ºC inside of the 6-well plates with or without QSIs (100 μM). The biofilms were washed twice before to remove loosely bound cells and stained with FITC-ConA for 15 min. Then the cells were washed twice with PBS to remove the excess stains and the biofilms were analyzed using CLSM (Zeiss L800, Zeiss, Tokyo, Japan) with the excitation and emission wavelength of 488 nm and 520 nm respectively.

### 4.5. SEM Analysis

The effect of QSIs on biofilm formation was visualized by scanning electron microscopy (SEM) as described by Singh et al., 2017 [43]. Biofilms of *P. aeruginosa* PAO1 were grown on glass coverslips in 6 well plates immersed in LB broths with or without QSIs (100 μM) for 24 h at 37 ºC. After incubation, the cover slips with biofilm were incubated with 2.5% glutaraldehyde for 20 min followed by 4% OsO4 in 0.1 M phosphate buffer for 30 min. Then the samples were dehydrated with a gradient ethanol series (10%–95%) for 10 min. The dried biofilms were coated with gold and visualized under SEM (VEGA 3, TESCAN USA, Inc., Kohoutovice, Czech Republic).

### 4.6. qRT-PCR Studies

*P. aeruginosa* PAO1 cells were grown in 1 mL LB medium with or without QSIs (100 μM) at 37 ºC for 24 h. Total RNA was extracted with the RNA isolation kit as per the manufacturer’s instructions (TIANGEN Biotech Co., Ltd., Beijing, China). Primers for *lasI, lasR, rhlI, rhlR,* and *proC* are listed in Table S1 and were synthesized by Sangon Biotech (Shanghai, China). The reverse transcription reaction was performed using a Prime Script RT Reagent Kit (TaKaRa, Tokyo, Japan) using total RNA as a template for at 37 °C for 15 min for three times (reverse transcription), and at 85 °C for 5 min (inactivation of reverse transcriptase), with a total volume of 20 μL. 

The qRT-PCR was performed with the SYBR^®^ Premix Ex TaqTM II Kit (TaKaRa, Japan) as per the manufacturer’s protocol. The cDNA was used as a template for qRT-PCR, and the total reaction system (20 μL) was made up of the following: 10 μL SYBR^®^ Premix Ex TaqTM II (2×), 0.8 μL forward primer, 0.8 μL reverse primer, 0.4 μL ROX Reference Dye (50×), 2 μL cDNA template, and 6 μL double-distilled H_2_O (ddH_2_O). The qRT-PCR was performed using Applied Bio systems QuantStudio™ 3 Real-Time PCR System (Applied Biosystems Inc., Waltham, MA, USA). The reaction conditions used were as follow: pre-denaturation at 95 °C for 30 s, followed by 40 cycles of denaturation at 95 °C for 5 s and annealing and extension at 60 °C for 30 s. proC was used as an internal reference [26]. The relative mRNA expression of all the genes was calculated using the 2−ΔΔCT method and the experiment was independently conducted in triplicates.

### 4.7. LasR-LBD Expression and Purification

All recombination procedures were performed based on LLHR (linear plus linear homologous recombination) of Red/ET recombineering established in our lab [44] (Figure S3), and related cloning primers were listed on Table S2. Proteins were purified based on the protocols by Lugo et al., 2017 [45] and the ÄKTA explorer system mount with Superdex 75 10/300 was used for further purification and buffer exchanging (Tm shift buffer: 50 mM K2HPO4/KH2PO4, 150 mM NaCl, 1mM DTT, pH 7.8, MST buffer: 50 mM Tris-Hcl 400 mM NaCl, 10 mM MgCl2 pH 7.8). After purification, SDS-PAGE analysis was done. 

### 4.8. Microscale Thermophoresis

The interactions between QSIs and LasR were analyzed with the concentration gradient of 50 μM of QSIs with 20 μM of LasR, which was labeled with Monolith NT™ Protein Labeling Kit RED-NHS before analysis. The optimized buffer for MST (50 mM Tris-HCl, 150 mM NaCl, 10 mM MgCl2, 0.05% Tween 20) and LED power (20%) was used. Analysis was performed on Monolith NanoTemper (NT) 115 (NanoTemper Technologies GmbH, Munich, Germany) and standard-treated 4 μL-volume glass capillaries were employed to measure the molecular interactions. The fluorescence intensity was obtained by the MST measurement fitted using NT 1.5.41 analysis software and the resultant *K*d values were given together with an error estimation [46].

### 4.9. Thermal Shift Assay (TSA)

The interaction of QSIs with LasR-LBD have been measured by Protein Thermal Shift Assay (Thermofisher Scientific, Waltham, MA, USA) as per manufacturers’ protocol. Using 20 μM the test compounds and 4 μM protein, TSA replicates were set and compared with the none-compound groups and none-protein groups. The thermal shift assay was conducted in Applied Bio systems QuantStudio- 3 Real-Time PCR System (Applied Biosystems Inc., Waltham, MA, USA). The protein melt reaction mixture was added to the wells of the 96-well PCR plate and then heated from 25 to 99 °C with a heating rate of 0.05 °C/S. The fluorescence intensity was measured with Ex/Em: 580/623 nm. Tm data were generated using the Boltzmann method and ΔTm was calculated by comparing the Tm values for LasR-LBD without ligand to those of LasR with ligand. Data were collected at 1 °C intervals from 25 °C through 99 °C on the Real Time PCR System and were analyzed using Protein Thermal ShiftTM Software v1.3 (Thermofisher Scientific (Applied Biosystems), Waltham, MA, USA).

### 4.10. Statistical Analysis

Graph pad prism software (v6.01) (GraphPad, San Diego, CA, USA) was used for statistical analysis. Multiple comparisons and one way ANOVA were applied wherever required. *P*-values (<0.05 and <0.001) were considered as statistically significant. All assays were performed in triplicates and the results were expressed as mean ± SD.

## 5. Conclusions

In view of the emergence of multi-drug resistant pathogens, it is necessary to develop novel strategies to find effective drugs against these multi-drug resistant pathogens. Since natural products continually play a major role in medicine and human health, virtual screening of natural products against the molecular drug targets will be a productive approach towards drug designing.

Overall results suggested that C7X, sappanol and butein are potential LasR-mediated quorum sensing inhibitors against *P. aeruginosa*, based on our in silico and in vitro assays, and further confirmation by CLSM and SEM studies. MST and Thermal shift assay confirmed bimolecular interactions of QSIs with LasR protein. Though C7X and sappanol show similar trends with C30, a known inhibitor, we speculate that butein induce misfolding and followed by aggregation of LasR protein by interacting with Tyr 47 present in L3 loop to allow inward moment of L3 loop. It is very clear that C7X and sappanol were down regulating the QS by potentially interaction with LasR protein of *P. aeruginosa* whereas further investigations are much warranted to ensure the mode of action of these QSIs in vivo.

Since homologues in LuxR-family are present in more than 100 g negative pathogens, these QSIs here may be developed as a broad spectrum anti-infective drug candidates. It is evident that starting with biological evaluation, gene expression studies and molecular interactions using MST and thermal shift assay will help us to get an in-depth understanding of these QSIs and their proposed mode of action. Considering the multi-drug resistance emergence in *P. aeruginosa*, it is essential to further evaluate these QSIs in animal models.

## Figures and Tables

**Table 1:** Docking results of quorum-sensing inhibitors against LasR, the quorum regulator of *P. aeruginosa*.

Name	Structure	GScore	H Bonds	Bond Forming Amino Acids
3OC12-HSL	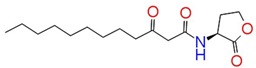	−6.456	5	Tyr 56 Trp 60 Asp 73 Ser 129 (2)
Catechin 7-xyloside (C7X)	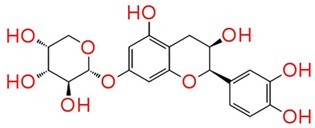	−13.508	4	Tyr 56 Tyr 64 Tyr 93 Leu 125
Sappanol	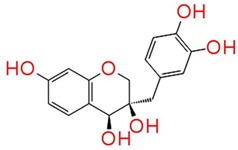	−10.964	4	Tyr 64 Thr 75 (2) Leu 125
Butein	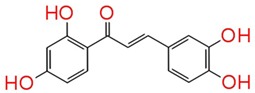	−9.245	3	Tyr 47 Thr 75 Ser 129
C30	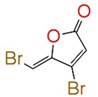	−4.244	1	Ser 129

## Supplementary Materials

Supplementary materials can be found at https://www.mdpi.com/1422-0067/21/6/2190/s1.

## Abbreviations

QS	Quorum sensing
QSIs	Quorum sensing inhibitors
C7X	Catechin-7-xyloside
MDR	Multi-drug resistant
AMR	Antimicrobial resistance
AIs	Autoinducers
AHL	Acyl homoserine lactone
LBD	Ligand binding domain
DBD	DNA-binding domain
3OC12-HSL	N-(3-oxododecanoyl)-L-homoserine lactone
C4-HSL	N-butanoyl-L-homoserine lactone
CLSM	Confocal laser scanning microscopy
SEM	Scanning electron microscope
MST	Microscale thermophoresis
*K*_D_	Dissociation constant
TSA	Thermal shift assay
Tm B	Boltzmann Tm
ECR	Elastin congo red
