# Supplementary material for: Attenuation of Pseudomonas aeruginosa Quorum Sensing by Natural Products: Virtual Screening, Evaluation and Biomolecular Interactions
Source: Int J Mol Sci. 2020 Mar 22;21(6):2190. doi: 10.3390/ijms21062190 (PMC7140002; doi:10.3390/ijms21062190)
Supplement: Supplementary file 1 [file ijms-21-02190-s001.pdf]

Supplementary Information

Attenuation of *Pseudomonas aeruginosa* Quorum Sensing by Natural Products: Virtual Screening, Evaluation and Biomolecular Interactions

Lin Zhong, Vinothkannan Ravichandran, Na Zhang, Hailong Wang, Xiaoying Bian *, Youming Zhang * and Aiying Li *

Helmholtz International Laboratory for Anti-Infectives, Shandong University-Helmholtz Institute of Biotechnology, State Key Laboratory of Microbial Technology, Shandong University, Qingdao 266237, P.R China; 201511767@mail.sdu.edu.cn (L.Z); vrvinothan@sdu.edu.cn (V.R.); 13342258573@163.com (N.Z) wanghailong@sdu.edu.cn (H.W).

* Correspondence: ayli@sdu.edu.cn (A.L), zhangyouming@sdu.edu.cn (Y.Z), bianxiaoying@sdu.edu.cn (X.B)

Table S1: Primers used in this study for qRT-PCR studies

Gene	Primers
lasI-5RT	CTA CAG CCT GCA GAA CGA CA
lasI-3RT	ATC TGG GTC TTG GCA TTG AG
lasR-5RT	ACG CTC AAG TGG AAA ATT GG
lasR-3RT	GTA GAT GGA CGG TTC CCA GA
rhlI-5RT	CTC TCT GAA TCG CTG GAA GG
rhlI-3RT	GAC GTC CTT GAG CAG GTA GG
rhlR-5RT	AGG AAT GAC GGA GGC TTT TT
rhlR-3RT	CCC GTA GTT CTG CAT CTG GT
proC- 5RT	GGC GTA TTT CTT CCT GCT GA
ProC- 3RT	CCT GCT CCA CTA GTG CTT CG

Table S2 Primers used for the LasR expression

Name	Bases
lasR-28a-5	TCATCATCATCACAGCAGCGGCCTGGTGCCGCGCGGCAGCATGGCCTTGGTTGACGGTTT
lasR-28a-3	CTTTGTTAGCAGCCGGATCTCAGTGGTGGTGGTGGTGGTGTCAGAGAGTAATAAGACCCAAATT

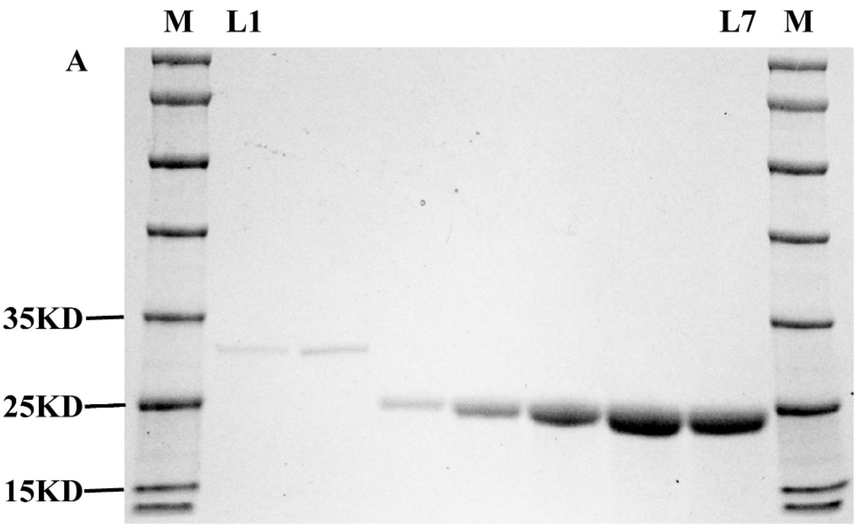

Figure S1: SDS-PAGE of purified protein for LBD (lane3-7) and (lane1-2) full length LasR.

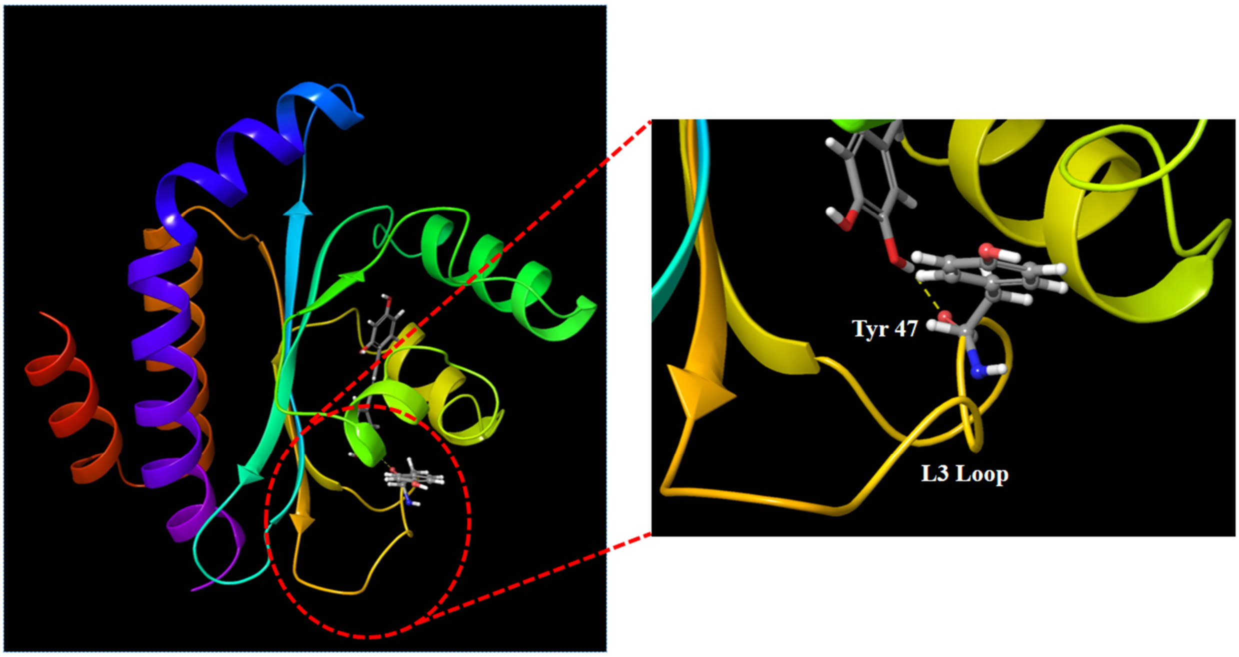

Figure S2: Interaction of B with LasR. Analysis showed that the bond length of hydroxyl group of B with carbonyl oxygen of Tyr 47 is 2.21Å.

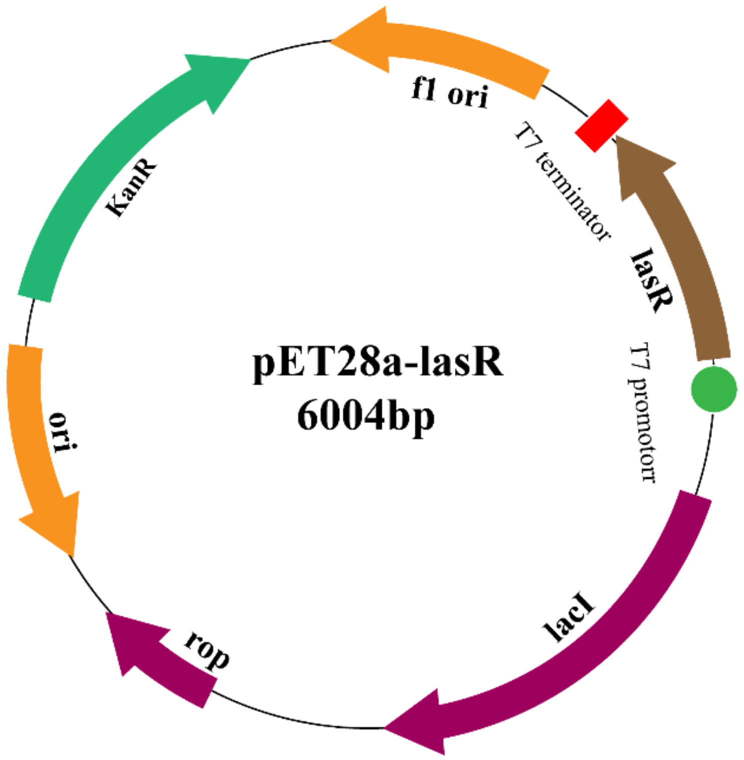

Figure S3: Construction map of pET28a-lasR plasmid.
